# Complete Genome Sequence of a Hyaluronate Lyase HysA- and HysB-Producing, Methicillin-Resistant Staphylococcus aureus Sequence Type 30, Staphylococcal Cassette Chromosome *mec* Type IVc Strain Isolated from Furunculosis in Japan

**DOI:** 10.1128/mra.00308-22

**Published:** 2022-08-16

**Authors:** Yuma Koizumi, Junzo Hisatsune, Atsushi Toyoda, Osamu Yamasaki, Jun Kaneko, Motoyuki Sugai

**Affiliations:** a Department of Orthodontics and Craniofacial Developmental Biology, Hiroshima University Graduate School of Biomedical and Health Sciences, Hiroshima, Japan; b Department of Antimicrobial Resistance, Hiroshima University Graduate School of Biomedical and Health Sciences, Hiroshima, Japan; c Antimicrobial Resistance Research Center, National Institute of Infectious Diseases, Tokyo, Japan; d Comparative Genomics Laboratory, National Institute of Genetics, Shizuoka, Japan; e Department of Dermatology, Shimane University Faculty of Medicine, Shimane, Japan; f Laboratory of Applied Microbiology, Department of Microbial Biotechnology, Graduate School of Agricultural Science, Tohoku University, Miyagi, Japan; University of Rochester School of Medicine and Dentistry

## Abstract

We report the complete genome sequence of Staphylococcus aureus strain JP025, which was isolated from a furunculosis sample from a Japanese patient. The strain carried two hyaluronate lyase genes, JP025*hysA* and JP025*hysB*, on the chromosome and was classified as sequence type 30.

## ANNOUNCEMENT

Staphylococcus aureus is a Gram-positive pathogen that is found in the nasal cavity and skin of humans. It causes various infectious diseases, such as pneumonia, septicemia, and food poisoning, and is especially known as the causative agent of skin diseases, such as furuncles and impetigo (1). Strain JP025 is a clinical isolate that was obtained from the pus of a furuncle from a Japanese patient (2). This study did not contain any personal information and was exempt from ethics committee review.

The JP025 strain, which had been stored as a 20% glycerol stock at −80°C, was cultured overnight on a Trypticase soy agar plate (Becton, Dickinson and Company, Franklin Lakes, NJ, USA). After incubation, a single colony was picked and incubated in Trypticase soy broth (Becton, Dickinson) at 37°C. After incubation, genomic DNA of JP025 was extracted using lysostaphin and the Qiagen Genomic-tip 20/G kit (Qiagen) according to the manufacturer's instructions. Short-read libraries were prepared for sequencing using KAPA HyperPlus kits (Nippon Genetics Co., Ltd., Tokyo, Japan) and run on an Illumina HiSeq sequencer. Short-read sequencing yielded 29,202,314 paired-end reads of 250 bp. For long-read sequencing, DNA shearing with a Covaris g-TUBE assembly (M&S Instruments, Inc.) was performed using the SMRTbell template preparation kit v1.0 (Pacific Biosciences [PacBio]) following the manufacturer’s protocols. The 15- to 50-kb library was selected using BluePippin (Sage Science). The prepared library was sequenced using P6-C4 chemistry with a single-molecule real-time (SMRT) Cell v3 on a PacBio RS II sequencer. Long-read sequencing resulted in 62,654 reads (average read length, 14,148 bp) after a quality check and adaptor trimming using fastp v0.20.0 with default parameters (3). *De novo* assembly using HGAP3 software with default parameters (https://github.com/ben-lerch/HGAP-3.0) produced two circular contigs, representing a chromosome and a plasmid. To correct sequencing errors, we mapped the Illumina reads to the assembled PacBio contigs using BWA-MEM v0.7.17 (https://github.com/lh3/bwa) and Pilon v1.24 (https://github.com/broadinstitute/pilon) with default parameters. In addition, we confirmed the circularization of this genome sequence using Unicycler v0.4.8 software with default parameters (4). The complete genome sequence was automatically annotated using DFAST with default parameters (https://github.com/nigyta/dfast_core) (5). We manually checked the start codons of coding sequences (CDSs) and gene names using In-silico Molecular Cloning genomics edition software (In Silico Biology, Inc., Japan) (6), and updated the product names.

The JP025 genome comprised a 2,794,309-bp chromosome and a 24,495-bp plasmid (pJP025), with G+C contents of 32.9 and 28.2%, respectively. The average coverage levels of the Illumina sequences for the chromosome and the plasmid were 2,538× and 5,422×, respectively. The chromosomal genome contained 2,602 predicted protein CDS, as well as 60 tRNA and 19 rRNA operons. pJP025 comprised 25 CDSs.

Genotyping showed that JP025 was classified as sequence type 30, coagulase type IV, *agr* type III, and staphylococcal cassette chromosome *mec* (SCC*mec*) type IVc using multilocus sequence typing (MLST) v2.19.0 (https://github.com/tseemann/mlst), BLAST v2.9.0 (7), and SCC*mec*Finder v1.2 (8) at the Center for Genomic Epidemiology (http://www.genomicepidemiology.org) with default parameters. Chromosomal DNA contained a Panton-Valentine leukocidin gene-carrying prophage. The JP025 genome also contained two hyaluronate lyase (*hys*) genes, encoding hyaluronic acid-degrading enzymes, at nucleotide positions 2,265,406 to 2,267,832 (locus tag SAJP025_20720) and 1,893.091 to 1,895,511 (locus tag SAJP025_17330). The amino acid sequence similarity between the two *hys* products was 87.1%, and the identity was 74.8%. By analogy to the nomenclature proposed for enterotoxin genes in S. aureus (9), the two *hys* genes were designated JP025*hysA* for SAJP025_20720 and JP025*hysB* for SAJP025_17330. Analysis of the entire genome sequence of JP025 will contribute to further understanding of furuncle formation by S. aureus.

### Data availability.

The complete genome sequence and the raw sequence data of S. aureus JP025 were deposited in DDBJ/EMBL/GenBank with accession numbers AP025683 and AP025684 for the chromosome and plasmid, respectively, and in the DDBJ Sequence Read Archive (DRA) with accession number DRA013850.
